# Nano-mechanical mapping of interdependent cell and ECM mechanics by AFM force spectroscopy

**DOI:** 10.1038/s41598-019-48566-7

**Published:** 2019-08-23

**Authors:** Prem Kumar Viji Babu, Carmela Rianna, Ursula Mirastschijski, Manfred Radmacher

**Affiliations:** 1 Institute of Biophysics, University of Bremen, Bremen, Germany; 2 Wound Repair Unit, Centre for Biomolecular Interactions Bremen, University of Bremen, Bremen, Germany

**Keywords:** Biophysics, Atomic force microscopy

## Abstract

Extracellular matrix (ECM), as a dynamic component of the tissue, influences cell behavior and plays an important role in cell mechanics and tissue homeostasis. Reciprocally, this three-dimensional scaffold is dynamically, structurally and mechanically modified by cells. In the field of biophysics, the independent role of cell and ECM mechanics has been largely investigated; however, there is a lack of experimental data reporting the interdependent interplay between cell and ECM mechanics, measured simultaneously. Here, using Atomic Force Microscopy (AFM) we have characterized five different decellularized matrices diverse in their topography, ECM composition and stiffness and cultured them with normal and pathological fibroblasts (scar and Dupuytren’s). We investigated the change in topography and elasticity of these matrices due to cell seeding, by using AFM peak force imaging and mechanical mapping, respectively. We found normal fibroblasts soften these matrices more than pathological fibroblasts, suggesting that pathological fibroblasts are profoundly influencing tissue stiffening in fibrosis. We detected different ECM composition of decellularized matrices used here influences fibroblast stiffness, thus highlighting that cell mechanics not only depends on ECM stiffness but also on their composition. We used confocal microscopy to assess fibroblasts invasion and found pathological fibroblasts were invading the matrices deeper than normal fibroblasts.

## Introduction

The extracellular matrix (ECM) is a structural scaffold made of non-cellular, fibrous and non-fibrous proteins that are able to influence cellular functions, tissue homeostasis and organ development^1^. Highly dynamic three-dimensional (3D) scaffolds provide environmental signals that directly regulate stem cell fate^2^. The ECM microenvironment mediates cell adhesion by providing the anchoring sequence tripeptide Arg-Gly-Asp (RGD) to cell transmembrane anchoring proteins such as integrins, which are an integral part of focal adhesions (FA)^3–5^. This molecular assembly connects cells and ECM via force pinpoints and contributes to cellular signaling such as mechano-sensation and mechanotransduction^6–8^. The mechanical signals provided by the ECM have an impact on cell mechanics and in response, cells have also huge impact on the ECM by modifying its composition, its architecture, and hence its mechanics and thus creating a reciprocal interplay between mechanics of cells and ECM. The bidirectional bio-chemical and bio-mechanical relationship between cells and ECM that is defined as dynamic reciprocity^9^ is a principal component of 3D tissues.

In order to evaluate cell and ECM mechanics in physiological conditions we have used the AFM, since it allows to record high resolution images and force-distance curves, often in forms of force maps, on biological samples in their physiological condition. The components that play a major role in determining cell and ECM elasticity are the actin cytoskeleton, collagen and elastin network structures^10–12^.

Under either normal or pathological conditions, tissues are subjected to various mechanical forces such as tension, compression and shear force that alter cell and ECM mechanics^13^. Several studies explored the influence of ECM topography and stiffness on cell mechanics^14,15^ and of cells on ECM topographic cues^16,17^ using AFM force spectroscopy. Previously, employing AFM nanoindentation, it has been reported that the leading edge of individual and collective cell migration deform the fibrillar collagen substrate and the substrate undergoes reversible non-linear strain stiffening^18,19^. The cellular proteolytic activity brings permanent changes to ECM composition, structure and mechanics^20,21^. Cellular traction forces induce permanent deformation of ECM collagen bundles suggesting a mechanical remodeling of the ECM by cells^22^. However, there is a lack of knowledge in following the permanent changes of ECM structure and mechanics by cells.

So far, cell elasticity was determined on individual ECM protein gels, mostly collagen^23^ and fibronectin^14^. The tremendous complexity of the 3D microenvironment makes the deep understanding of the mechanical reciprocity between cells and ECM very complicated which in turn results in difficulties to follow the changes in cell and ECM mechanics at the same time. To overcome this issue, there is a strong need to employ more complex and heterogeneous matrices able to provide the full range of signals, where cells are exposed to, in near physiological conditions.

The emerging trend using decellularized samples provides cells with an enormously rich and tunable chemical and mechanical microenvironment. With the already reported decellularization protocols^24^, a natural ECM scaffold can be prepared through chemical, physical or enzymatic procedures. Interestingly, decellularized tissues offer a more physiologically relevant microenvironment to cells than 3D cultures using single or only a very limited variety of ECM components^25^. Different decellularization protocols preserve the chemical and mechanical integrity of the native ECM scaffold in order to study ECM micromechanics. In order to study their mechanical properties, AFM indentation experiments were set up to probe the decellularized lung ECM micromechanics and to study the anatomical specific regional heterogeneities^20^.

Here, we characterised five matrices that will be further termed “decellularized”: human DED (de-epidermized dermis), human Amnion (allogenic, acellular), Epiflex (allogenic, acellular) and porcine XenoDerm (xenogenic, acellular), and MatriDerm (alloplastic, artificial). Each one differs in composition, topography and stiffness. Some of these decellularized matrices are seeded with cells and often used in wound repair and organ regeneration^26–28^. We have chosen these matrices in order to study the role of fibroblasts in ECM maintenance by mechanical and topographical evaluation. DED was prepared by us in a physical decellularization procedure^12^, whereas the others are commercially purchased. In general, ECM secretion, deposition and degradation are highly regulated by fibroblasts^29,30^. For this study, three types of primary fibroblasts of different origin (normal fibroblasts from normal healthy skin and scar fibroblasts from cutaneous scar tissue and Dupuytren’s fibroblasts from the palmar fascia of the same patient with Dupuytren’s disease) were seeded onto these matrices to monitor the interdependent cell and ECM mechanics. As the fibroblasts-scar and Dupuytren’s are extracted respectively from the pathological environment- scar region and cord and nodule of palmar fascial region, we termed them as pathological fibroblasts. The aim of this work was to visualize and observe changes in ECM structure and mechanics caused by cells. To achieve this aim, we recorded AFM peak force tapping mode images and mechanical force maps on the five decellularized matrices for different categories: bare matrix, matrix populated by cells, and the same matrices after cell removal for each fibroblast (normal, scar and Dupuytren’s). This setup allows to probe quantitatively the ECM topography and mechanics in all different categories and allows to study the effect of fibroblasts on ECM features. We found that fibroblasts had a strong effect on decellularized matrices topography and stiffness and were able to modify the matrix, causing a change in mechanical properties before and after cell culture, even after the cells were removed. Also, we hypothesized that decellularized matrices provide the natural and biomimetic microenvironment to study both cell and ECM mechanics. Furthermore, we show that the Young’s modulus of fibroblasts differs when fibroblasts are seeded on different matrices, possibly due to the peculiar matrix composition, thus implicating the dependence of cell mechanics to ECM composition. Finally, we were able to obtain z stack images using confocal fluorescence microscopy, demonstrating the degree of cell invasion into decellularized matrices by three different fibroblast types.

## Results

### General setup and characterisation of decellularized matrices

**Figure 1:**
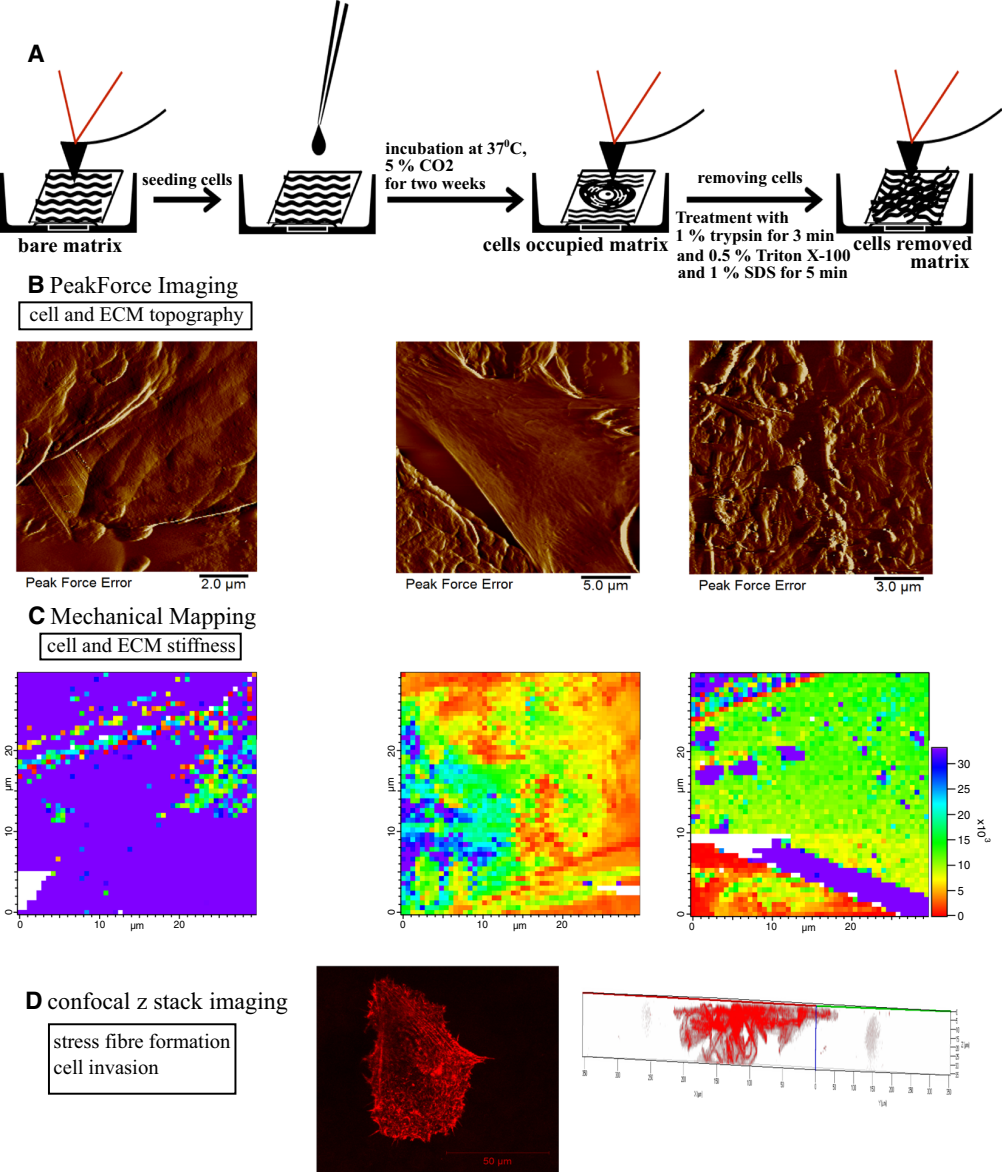
Schematic representation of the overall experimental setup for cell and ECM topography and mechanics investigation on decellularized matrix. (**A**) Decellularized matrices were seeded with fibroblasts (normal, scar or Dupuytren’s) and incubated at 37 °C for two weeks. Thereafter, cells were removed by chemical treatment (1% trypsin, 0.5% Triton X-100 and 1% SDS) to follow the changes in matrix topography and mechanics. (**B**) AFM Peak force imaging shows the topography of native Epiflex matrix, matrix with cells and matrix after removal of cells. (**C**) AFM mechanical mapping illustrates the stiffness of bare Epiflex matrix, matrix with cells and matrix after removal of cells (the maps have the same scale and show values of Young Modulus, Pa). (**D**) z stack imaging from confocal microscopy displays the stress fibre formation in fibroblasts and also creates 3D maps that evaluate the degree of cell invasion into the decellularized matrices.

The overviews of the different steps of the experiment are schematically shown in Fig. 1A. First, AFM measurements were performed on bare matrices, and then cells were seeded on the matrices and cultured for two weeks. This was followed by repeated AFM measurements of these matrices populated with cells. Finally, cells were removed and bare, but possibly restructured or modified matrices were investigated again by AFM. By obtaining PeakForce Tapping AFM images (Fig. 1B) and mechanical maps (Fig. 1C) at each step, we were able to observe changes in ECM topographical and mechanical properties and also cell stiffness within the same matrix, investigating the reciprocal effect of cell and ECM mechanics on each other. Finally, by performing confocal microscopy z stack imaging for cells on matrices (Fig. 1D), we were able to study fibroblast invasion into the matrix, visualizing the stress fibre network and relating this information to the cell stiffness measurements.

**Figure 2:**
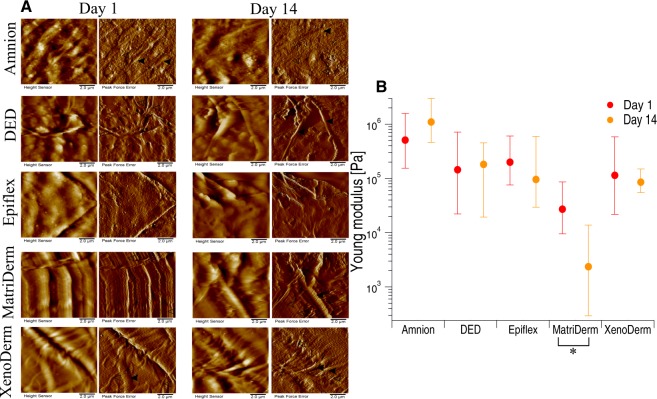
Influence of liquid environment on decellularized matrix topography and mechanics. (**A**) Height and Peak force error images from PeakForce Tapping imaging show no larger effect of liquid between day 1 and day 14 on Amnion and XenoDerm matrix topography. In MatriDerm, some collapsed fibres were observed along with well aligned fibres on day 14. Random fibres were running along DED and plain surfaces were seen in Epiflex. (**B**) The median of Young’s modulus values were obtained from force maps that enable the assessment of the stiffness of decellularized matrices between day 1 and day 14. There is a change in Young’s modulus value observed in Epiflex, MatriDerm and XenoDerm matrices but not in DED or Amnion on day 14 which is due to the impact of liquid environment. *Filled* and *open arrow heads* indicates the thick and thin fibres, respectively. Statistical results are reported in Materials and Methods section.

Since in our experimental setup the matrices were incubated for 14 days with cells prior to AFM measurements, we decided to test the bare matrix stiffness and topography at day 1 and day 14, i.e. the first day and after 14 days of culture in PBS in the incubator (as a control for other samples, which were incubated with cells for up to 14 days). Figure 2A shows the height and peak force error images of decellularized bare matrices (without cells) imaged in PBS on day 1 and day 14 (left and right panel, respectively). Height and peak force error images from AFM Peak Force Tapping Imaging show the respective overall topography and fine details such as fibre thickness of the decellularized matrices. The topographical and mechanical characterisation of the five decellularized matrices at different times gives the possibility to track the influence of liquid environment on ECM topography and stiffness. Height and peak force error images of Amnion matrices show the presence of cross-linked thick and thin fibres with some other ECM remnants. Thickness of thick and thin fibres varied between 0.5–0.7 µm and 0.15–0.2 µm, respectively The microscopic appearance of DED and Epiflex matrices shows no proper fibre alignment but irregular blobs and corrugated surfaces with few fibres. Traces of thin fibres of 0.25–0.3 µm thickness were observed in DED matrix. A well oriented fibre alignment was observed in MatriDerm and XenoDerm. MatriDerm images show thin fibres of 0.125–0.275 µm thickness running parallel to each other and sometimes tailored structures. XenoDerm images show large thick fibres of fibre thickness 0.9–1.4 µm and thin fibres of 0.235–0.375 µm thickness, which are bundled closely together. Although few fibres (0.45–0.5 µm thickness) running along the matrices were observed in DED after 14 days in PBS, there was no particular fibre alignment or pattern. Despite the small topographic differences due to measurements taken on different positions in the Epiflex and MatiDerm matrices, there was no change in pattern or disruption or misalignment of fibres observed between day 1 and day 14. Mostly, no larger variability in Amnion and XenoDerm topography were found between day 1 and day 14. Additionally, no effect of aging of matrices observed microscopically and also macroscopically no change in texture of matrices witnessed.

We also recorded high resolution force maps (each map = 50 × 50 = 2500 force curves) in at least 10–12 different positions on the decellularized matrices on day 1 and day 14. These force maps recorded on the decellularized matrices showed ECM fibers (indicated by *blue arrows* in Supplementary Fig. 1A). From the force measurements the mechanical properties are obtained by fitting each force curve with the Hertz model to obtain and plot the respective median Young’s modulus values (Fig. 2B). In some cases, we could observe a decrease in Young’s modulus after 14 days, especially in Epiflex (from 199.5 kPa to 95.8 kPa – a two-fold decrease), MatriDerm (Young modulus significantly changed from 27.1 kPa to 2.3 kPa – a ten-fold decrease) and XenoDerm (from 114.2 kPa to 85.3 kPa). An explanation for this discrepancy could be the influence of the liquid environment over the incubation time of two weeks. In contrast, for DED there was no significant change in Young’s modulus apparent (144.4 kPa on day 1 and 181.4 kPa on day 14). In contrast to macroscopic appearance as a shiny membrane, Amnion was characterized to be a “super stiff” ECM substrate. We could not quantify the Young’s modulus due to the soft cantilever used. The quoted values (0.5 MPa on day 1 and 1.09 MPa on day 14) shown in Fig. 2B reflects the comparatively softest areas (calculated from fewer force curves- Supplementary Fig. 1B) within the sample and should not be over interpreted. Together, our results show that the liquid environment has no significant effect on the structures of the decellularized matrices except for DED and Epiflex and has a large effect on the mechanics of MatriDerm over a period of 14 days. As a consequence, any notable effect seen after the incubation with cells was due to the presence of the cells and not exclusively an effect of the liquid environment.

### Changes in decellularized matrices topography and mechanics by fibroblast

The structure and mechanics of tissues are constantly altered biochemically as well as by cellular traction forces, which results in permanent topographical and mechanical changes of the extracellular matrix microenvironment. Earlier reports observed a reversible nonlinear strain stiffening^18^ and irreversible plasticity^22^ of collagen ECM networks due to cell traction forces. In order to measure the resulting ECM topographical and mechanical changes induced by cellular activity, three different fibroblast types derived from different sites of the same patient (normal, scar and Dupuytren’s fibroblast) were grown on the five different decellularized matrices used here. As presented above, we monitored the effect of liquid environment on the topography and stiffness of decellularized matrices. In a similar way, matrices were topographically imaged and mechanically mapped (at least 10 different positions) before cell culture, with cells seeded on them and finally after removing cells.

**Figure 3:**
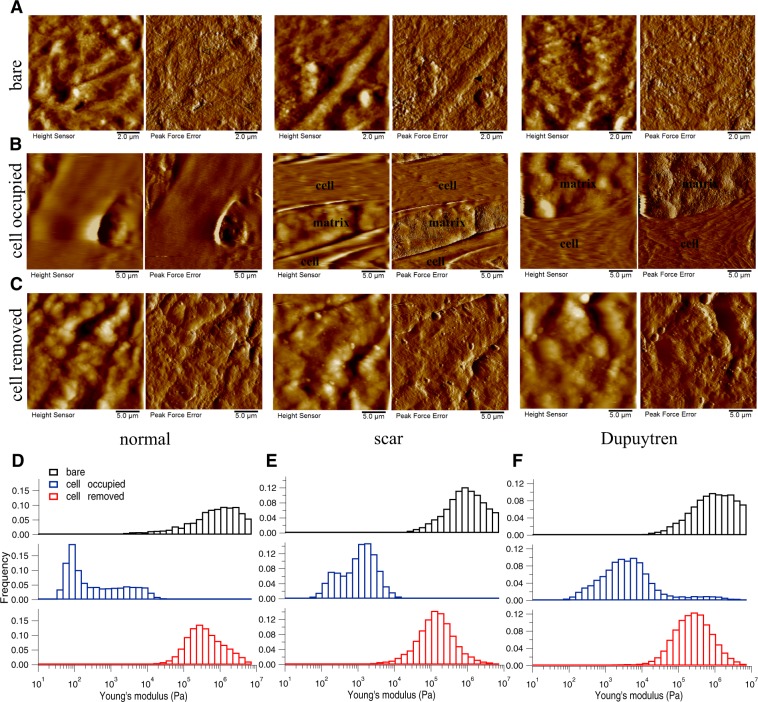
Changes in decellularized matrix topography and mechanics assessed in Amnion. (**A**) Height and PeakForce error images of the bare matrix show the presence of very thin and thick irregular fibres in bare matrix. (**B**) After seeding and culture of normal, scar and Dupuytren’s fibroblasts for two weeks, height and PeakForce error images show the presence of fibroblasts on Amnion matrix. (**C**) After removing the cells, height and PeakForce error images show the disappearance of fibres. Histograms of Young’s modulus values of bare (*black bar*), cell-occupied (*blue bar*), and cell- removed Amnion matrix (*red bar*), for normal (**D**), scar (**E**) and Dupuytren’s fibroblasts (**F**). The shifts in the histograms show the change in Amnion matrix mechanics effected by all three fibroblast types. The respective medians of Young’s modulus values are shown in Supplementary Fig. 2A. *Filled* and *open arrow heads* indicates the thick and thin fibres, respectively.

**Figure 4:**
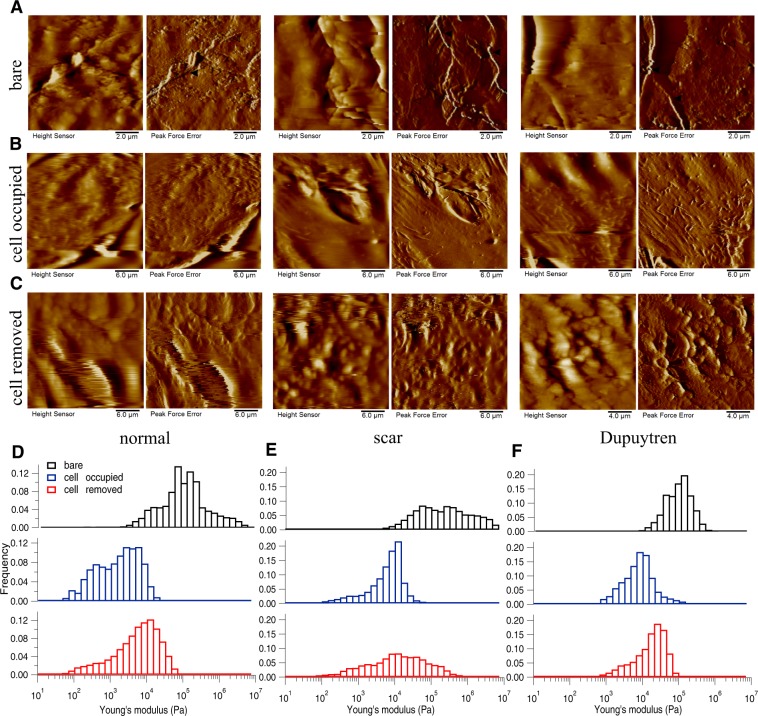
Changes in decellularized matrix topography and mechanics assessed in DED. (**A**) Height and PeakForce error images of bare matrix show the presence of irregular structures of ECM components in the bare matrix. (**B**) After cell culture for two weeks, height and PeakForce error images show the matrix with normal, scar or Dupuytren’s fibroblasts. (**C**) After removing the cells, height and PeakForce error images show the matrix topography. Histograms of Young’s modulus values of bare (*black bar*), cell-occupied (*blue bar*), and cell-removed DED matrix (*red bar*), for normal (**D**), scar (**E**) and Dupuytren’s fibroblasts (**F**). The shifts in the histograms show the change in DED matrix mechanics by all three fibroblast types. The respective medians of Young’s modulus values are shown in Supplementary Fig. 2B. *Filled* and *open arrow heads* indicates the thick and thin fibres, respectively.

**Figure 5:**
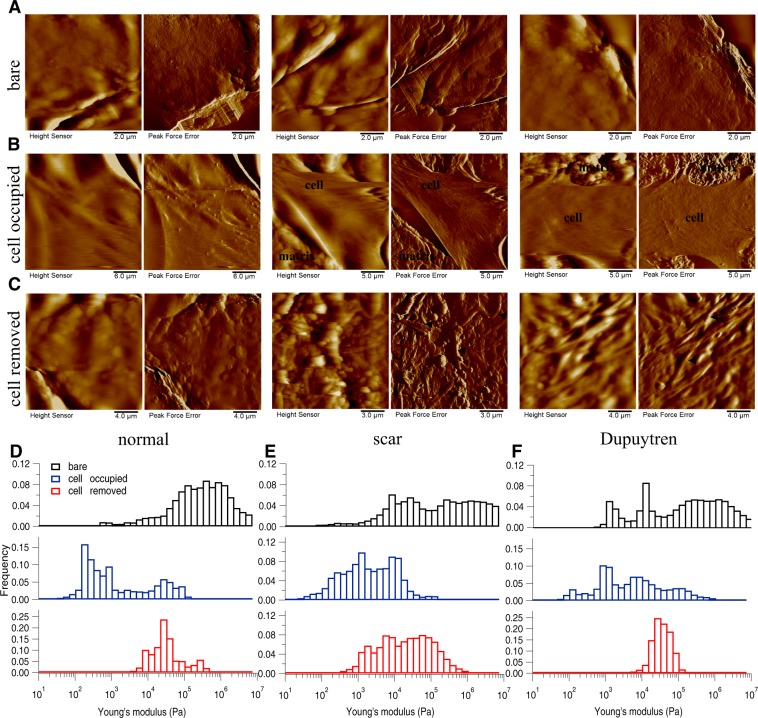
Changes in decellularized matix topography and mechanics assessed in Epiflex. (**A**) Height and PeakForce error images of bare matrix show the presence of bigger blobs with some very thin fibres in the bare matrix. (**B**) After culture the three fibroblast types for two weeks, height and PeakForce error images show the topography of matrix with respective cells. (**C**) After removing the cells, height and PeakForce error images shows the change in matrix topography. Histograms of Young’s modulus values of bare (*black bar*), cell-occupied (*blue bar*), and cell-removed Epiflex matrix (*red bar*), for normal (**D**), scar (**E**) and Dupuytren’s fibroblasts (**F**). The shifts in the histograms show the change in Epiflex matrix mechanics by all three fibroblast types. The respective medians of the Young’s modulus values are shown in Supplementary Fig. 2C. *Open arrow heads* indicates the thin fibres.

**Figure 6:**
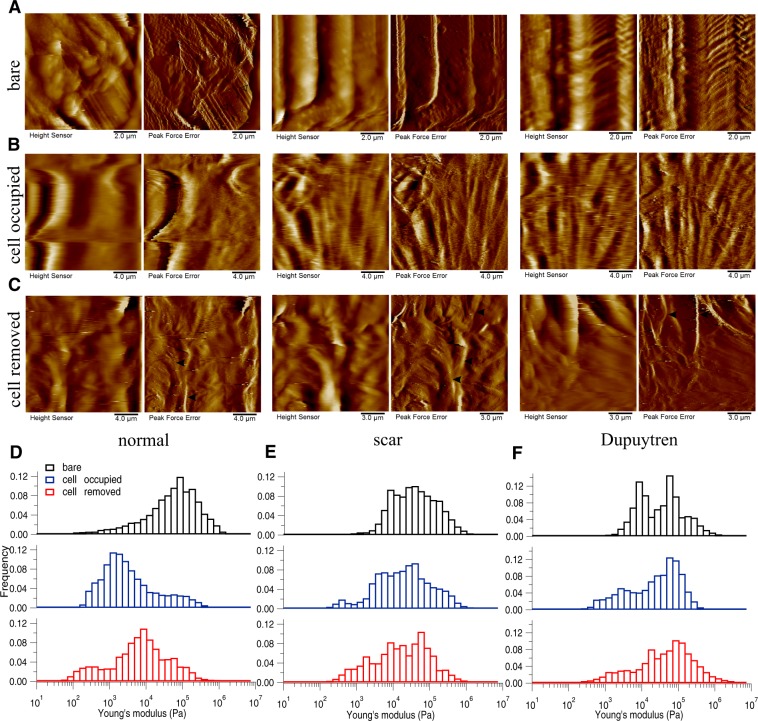
Changes in decellularized matix topography and mechanics assessed in MatriDerm. (**A**) Height and PeakForce error images of bare matrix show the presence of tailored and more dense fibres running along the bare matrix. (**B**) After fibroblast culture for two weeks, height and PeakForce error images show the matrix with cells. (**C**) After removing the cells, height and PeakForce error images show the change in matrix topography. Histograms of Young’s modulus values of bare (*black bar*), cell-occupied (*blue bar*), and cell-removed MatriDerm matrix (*red bar*), for normal (**D**), scar (**E**) and Dupuytren’s fibroblasts (**F**). The shifts in the histograms show the change in MatriDerm matrix mechanics by all three fibroblast types. The respective medians of the Young’s modulus values are shown in Supplementary Fig. 2D. *Filled* and *open arrow heads* indicates the thick and thin fibres, respectively.

**Figure 7:**
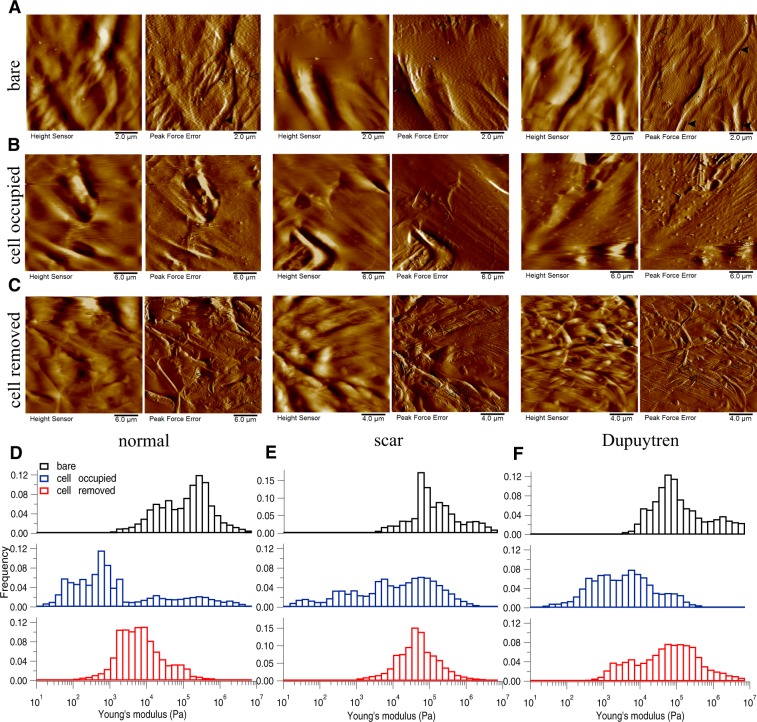
Changes in decellularized matix topography and mechanics assessed in XenoDerm. (**A**) Height and PeakForce error images of bare matrix show the presence of thick fibres running along the bare matrix. (**B**) After culturing normal, scar or Dupuytren’s fibroblasts for two weeks, height and PeakForce error images show the matrix with cells. (**C**) After removing the cells, height and PeakForce error images show the change in matrix topography. Histograms of Young’s modulus values of bare (*black bar*), cell-occupied (*blue bar*), and cell-removed XenoDerm matrix (*red bar*), for normal (**D**), scar (**E**) and Dupuytren’s fibroblasts (**F**). The shifts in the histograms show the change in XenoDerm matrix mechanics by all three fibroblast types. The respective medians of the Young’s modulus values are shown in Supplementary Fig. 2E. *Filled* and *open arrow heads* indicates the thick and thin fibres, respectively.

As stated above, the topography of all five decellularized matrices before adding cells was recorded using PeakForce Tapping AFM mode and the corresponding height and peak force error images are shown in Fig. 3A (Amnion), in Fig. 4A (DED), in Fig. 5A (Epiflex), in Fig. 6A (MatriDerm) and in Fig. 7A (XenoDerm). From the topographic images of Amnion, DED and XenoDerm, we did not find any larger structural differences within the three independent experiments of individual matrices (before adding cells) proving that matrices were quite homogenous within the same category. In contrast, three independent experiments on individual Epiflex and MatriDerm matrices showed some variability in their topography. In Epiflex (Fig. 5), two distinct regions were observed: corrugated surface and very thin fibres (0.078 µm thickness) running under the corrugated surface. In some specimen, only the corrugated surface was observed. In MatriDerm, thick fibres were seen with different geometries like tailored and totally compacted shapes. Histograms from AFM mechanical maps for bare matrix before adding fibroblasts were plotted for each matrix, i.e. Amnion (Fig. 3D–F), DED (Fig. 4D–F), Epiflex (Fig. 5D–F), MatriDerm (Fig. 6D–F) and XenoDerm (Fig. 7D–F) (black bar plots). Their respective medians of Young’s modulus values were plotted in Supplementary Fig. 2A–E (black filled circles) and listed in Table 1. A large difference in the modulus values was observed for all three independent experiments with regard to each decellularized matrix: Amnion (mostly infinite pascal for all three independent experiments- 760.6 kPa, 751.3 kPa and 851.7 kPa), DED (65.7 kPa, 171.6 kPa and 73 kPa), Epiflex (250.6 kPa, 139 kPa and 101.6 kPa), MatriDerm (51.4 kPa, 29.8 kPa and 28.1 kPa) and XenoDerm (119.2 kPa, 82.9 kPa and 70.8 kPa) which show the heterogeneity within each decellularized matrix mechanical properties but are not significant.

**Table 1:** The median (bold values) Young’s moduli (kPa) values of bare matrices before seeding with normal, scar or Dupuytren’s fibroblasts.

Matrix	Amnion			DED			Epiflex			MatriDerm			XenoDerm		
Fibroblast type	25th	Median	75th	25th	Median	75th	25th	Median	75th	25th	Median	75th	25th	Median	75th
normal	552.50	**760.67**	1383.11	37.74	**65.72**	83.95	182.60	**250.62**	557.33	36.21	**51.4**	78.84	94.79	**119.25**	165.97
scar	445.19	**751.33**	1131.38	124.71	**171.64**	461.16	125.86	**139.03**	934.30	19.67	**29.86**	58.54	35.25	**82.93**	185.75
Dupuytren	563.50	**851.72**	1582.59	35.54	**73.06**	46.18	94.13	**101.62**	487.37	20.41	**28.17**	34.80	39.73	**70.89**	321.44

**Figure 8:**
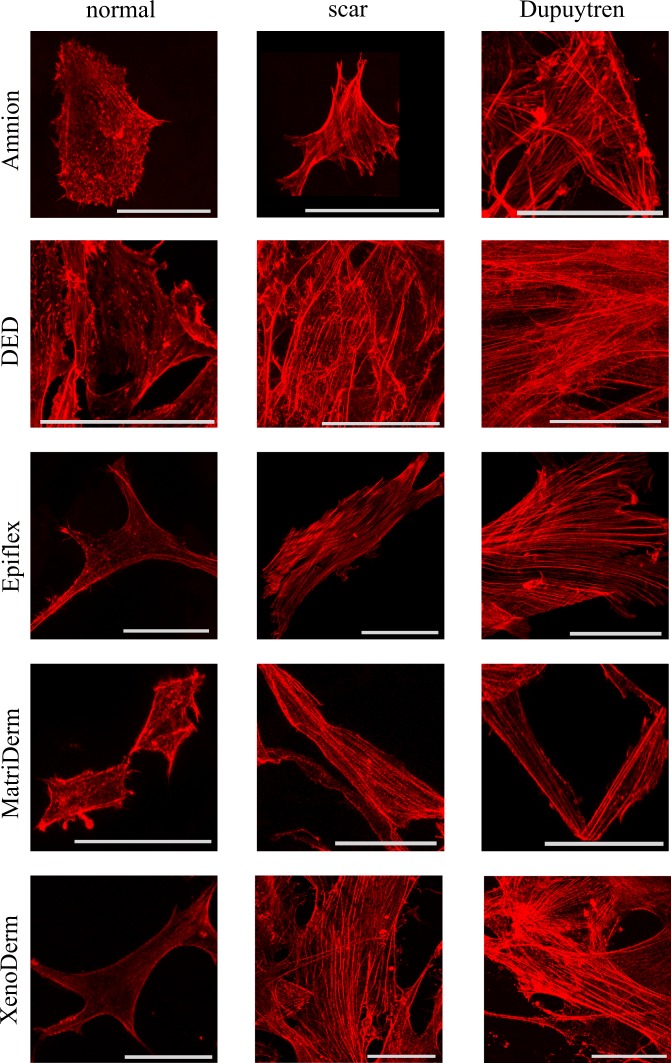
Confocal images of normal, scar and Dupuytren fibroblasts seeded on different matrices. Fibroblast stress fibre formation in decellularized matrices is assessed by rhodamine phalloidin actin fluorescent staining. Fluorescence images show the presence of thick stress fibres in pathological fibroblasts (scar and Dupuytren’s) that results in higher values of cell mechanics compared to normal fibroblasts that express less or no stress fibres on all decellularized matrices and therefore impress as softer. Scale bar are 50 μm.

Three fibroblast cell types were grown on these five decellularized bare matrices for two weeks in order to evaluate the topography and stiffness of our cell populated matrices. Amnion (Fig. 3B), DED (Fig. 4B), Epiflex (Fig. 5B), MatriDerm (Fig. 6B) and XenoDerm (Fig. 7B) shows the overall topography of cells on decellularized matrices in which all three fibroblasts remains viable and exhibit regular cell shape and spread and cytoskeletal organisation. In some of the PeakForce AFM micrographs, we were able to show both cell and ECM topography and revealed the change in ECM topography (Figs 3B and 5B). Histograms from AFM mechanical maps for cells occupied matrix of all three fibroblasts were plotted for each matrix - Amnion (Fig. 3D–F), DED (Fig. 4D–F), Epiflex (Fig. 5D–F), MatriDerm (Fig. 6D–F) and XenoDerm (Fig. 7D–F) (blue bar plots). These histograms showed a shift in the cell occupied matrix bar (blue) comparative to bare matrix bar (black). To measure quantitatively the mechanical heterogeneity of cell and ECM stiffness, the median of Young’s modulus values was plotted in Supplementary Fig. 2A–E- blue filled circles and listed in Table 2. Comparatively, the median Young’s modulus of normal fibroblast populated matrices (0.2 kPa on Amnion, 1.5 kPa on DED, 0.3 kPa on Epiflex, 1.9 kPa on MatriDerm and 0.4 kPa on XenoDerm) are smaller than those of scar (0.9 kPa on Amnion, 5.9 kPa on DED, 1.4 kPa on Epiflex, 11.5 kPa on MatriDerm and 1.8 kPa on XenoDerm) and Dupuytren’s fibroblasts (2.9 kPa on Amnion, 6.7 kPa on DED, 3.3 kPa on Epiflex, 13.2 kPa on MatriDerm and 2 kPa on XenoDerm) populated matrices. Here, Young’s modulus median values reflect both cell and ECM stiffness properties as some force maps were recorded on cell and ECM regions. Fluorescence images (Fig. 8) show the stress fibre network of normal, scar and Dupuytren’s fibroblasts on Amnion, DED, Epiflex, MatriDerm and XenoDerm. On all matrices, we observed that normal skin fibroblasts exhibited no stress fibres than scar or Dupuytren fibroblasts, which correlates to their respective lower and higher Young’s modulus values.

**Table 2:** The median (bold values) Young’s moduli (kPa) values of cell-occupied matrices when seeded with normal, scar or Dupuytren’s fibroblasts.

Matrix	Amnion			DED			Epiflex			MatriDerm			XenoDerm		
Fibroblast type	25th	Median	75th	25th	Median	75th	25th	Median	75th	25th	Median	75th	25th	Median	75th
normal	0.09	**0.16**	1.43	1.08	**1.49**	2.10	0.16	**0.33**	0.73	1.10	**1.92**	5.55	0.31	**0.41**	0.67
scar	0.61	**0.94**	0.85	3.51	**5.94**	3.32	1.01	**1.45**	5.01	7.89	**11.48**	20.88	1.53	**1.79**	14.55
Dupuytren	1.94	**2.96**	5.31	3.44	**6.76**	4.30	2.47	**3.27**	11.73	10.66	**13.2**	22.62	1.51	**2.03**	5.49

To determine if the fibroblast culture could alter the topography and mechanics of the decellularized matrices themselves, we repeated the measurements after removing the cells by chemical treatment (see Materials and Methods). Height and Peakforce error images (Figs 3–6 and 7C) show enormous differences between the decellularized matrices, before cell seeding and after cell removal. From AFM imaging, we were not able to see any cellular debris or remnants on the matrices. With regard to Amnion (Fig. 3C), we found no ECM fibres after cell removal and the matrix structure was more corrugated from all three fibroblast removed matrices. For DED matrix after fibroblasts removal (Fig. 4C), it was difficult to find any difference in ECM topography, we could only appreciate a decrease in the number of fibres and the presence of bigger blobs after cell removal. Despite of the corrugated surface in Epiflex (Fig. 5C), we could see thicker and uneven fibres (0.7–0.9 µm thickness) exclusively on samples that were previously populated with scar or Dupuytren’s fibroblasts, whereas there were only smaller blobs when matrices had been seeded with normal fibroblast. In case of MatriDerm, thin fibres initially found in the bare matrix (Fig. 6A) disappeared after cell removal (Fig. 6C), giving place to more irregularly aggregated and thick fibres (1.3–1.4 µm thickness) in all three fibroblast removed matrices. In XenoDerm (Fig. 7C), the thick and bundled fibres were disrupted to form irregular and disarranged thin fibres (0.2–0.4 µm thickness) after fibroblasts removal.

These topographical modifications after fibroblast culture were accompanied by a softening of the matrices. In fact, from the mechanical mapping data, we observed a shift in the histograms bar compared to bare (black) and cell occupied (blue) matrix in Amnion (Fig. 3D–F), DED (Fig. 4D–F), Epiflex (Fig. 5D–F), MatriDerm (Fig. 6D–F) and XenoDerm (Fig. 7D–F) (red bar plots) matrices after cell removal, for all three fibroblast types. The corresponding median of Young’s modulus values for all fibroblast-removed matrices were plotted in Supplementary Fig. 2A–E (red filled circles) and listed in Table 3. The median Young’s modulus values of bare, cell-populated and cell-removed of all five decellularized matrices that were seeded with normal or pathological (scar and Dupuytren’s) fibroblasts, respectively, are presented in Tables 1, 2 or 3, accordingly. The comparative change in Young’s modulus values of bare and cell-removed matrices reveals the extent of matrix mechanics and remodelling induced by fibroblasts. Bare Amnion is a very stiff matrix and shows high Young’s modulus values (MPa) – even the calculated Young’s modulus values (Table 1) from fewer force curves which are recorded in comparatively softest areas within the stiff matrix. Mostly, all force curves evaluated from cell-removed Amnion matrices show a gradual decrease in Young’s modulus. The shift in the peak of the histograms (Figs 3–6 and 7D–F) clearly shows the unique changes in ECM elasticity before adding cells (black bar), with cells (blue bar), and after removing the cells (red bar). In particular, normal fibroblasts soften the matrices by a factor of 10 compared to scar and Dupuytren’s fibroblasts in all matrices contrasting scar and Dupuytren’s fibroblasts that have a minor effect on matrix softening. This important finding is highlighted by biophysical measurements with MatriDerm. Here, we observed the median Young’s modulus values from bare matrices before adding cells (29.86 kPa and 28.17 kPa) and cell-removed matrices (14.15 kPa and 35.4 kPa) only when pathological (scar and Dupuytren’s) fibroblasts were seeded on MatriDerm matrices (Fig. 6). Taking into account the influence of liquid on MatriDerm mechanics (Fig. 2B), these pathological fibroblasts greatly maintained the matrix stiffness. Finally, these results clearly demonstrate that fibroblasts reciprocally influence their ECM microenvironment topography and mechanics in general and that the pathological scar and Dupuytren’s fibroblasts may play an important role in fibrosis by tissue stiffening in particular.

**Table 3:** The median (bold values) Young’s moduli (kPa) values of cell-removed matrices after seeding with normal, scar or Dupuytren’s fibroblasts.

Matrix	Amnion			DED			Epiflex			MatriDerm			XenoDerm		
Fibroblast type	25th	Median	75th	25th	Median	75th	25th	Median	75th	25th	Median	75th	25th	Median	75th
normal	109.16	**228.73**	261.16	3.52	**5.18**	6.49	10.18	**23**	16.82	4.14	**5.82**	11.62	2.96	**4.89**	8.09
scar	578.42	**108.15**	115.15	7.43	**9.83**	27.14	12.34	**16.49**	41.83	10.51	**14.15**	33.42	20.71	**37.1**	50.40
Dupuytren	110.82	**193.75**	240.72	9.25	**16.41**	11.05	8.82	**27.22**	14.53	25.82	**35.4**	61.27	34.40	**42.57**	113.11

### ECM composition dependent cell stiffness

**Figure 9:**
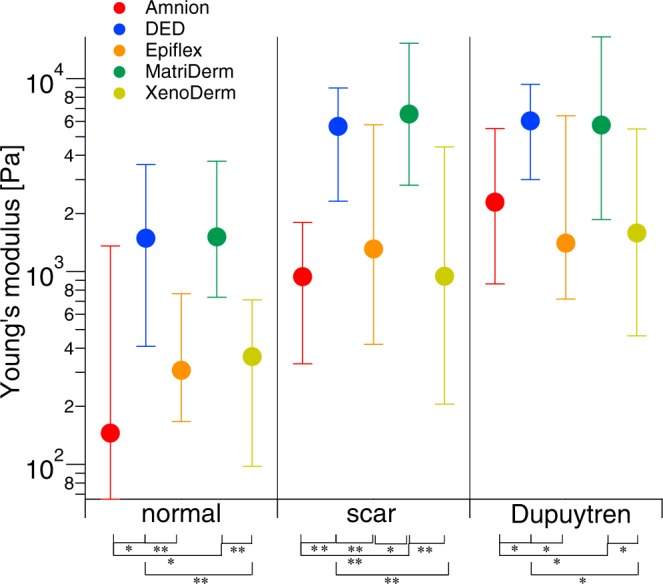
Cell stiffness is influenced by ECM composition, measured with AFM. After filtering the values of native matrices Young’s modulus values from cell-occupied matrices, the median of Young’s modulus values of normal, scar and Dupuytren’s fibroblasts shows that the composition of the different decellularized matrices, e.g. Amnion, DED, Epiflex, MatriDerm or XenoDerm, determines the fibroblast mechanics. The respective medians of the Young’s moduli values were presented in Table 4. Statistical results are reported in Materials and Methods section.

Many studies have reported that ECM stiffness largely influences many cell characteristics such as spreading, adhesion and mechanics^14,15,31,32^. However, the relevance of ECM composition on cell mechanics has been scarcely explored. Here, we studied the mechanical properties of normal, scar and Dupuytren’s fibroblast on five different decellularized matrices. In order to evaluate the mere cell stiffness, the mechanical maps obtained from matrices after plating cells were carefully analyzed and treated to eliminate the stiffness contribution of the matrix. As shown in Supplementary Fig. 3, the slope of force curves of the matrices greater than 0.375 were filtered out from the mechanical maps thus resulting in the force curves that only identify cell stiffness (see Materials and Methods section). This value was identified by carefully measuring the slope on matrix and cells from each force map which is able to distinguish cell and matrix regions in better resolution force maps and finding a threshold value between the two. The median Young’s modulus (Fig. 9) of normal, scar and Dupuytren’s fibroblasts and their respective values in Table 4 show that they were softer on three matrices, i.e. Amnion, Epiflex and XenoDerm. In contrast, all three fibroblast cell types were stiffer when seeded on DED and MatriDerm. This mechanical difference could be possibly due to different ECM composition of these decellularized matrices, since their stiffness is quite comparable, except for Amnion (Fig. 2B). In fact, all decellularized matrices used here consist of different compositions of collagen, elastin and other varying ECM structural and functional components^26,27,33–35^. In each decellularized matrix that was seeded with cells, even after exclusion of the matrix stiffness, the pathological (scar and Dupuytren’s) fibroblasts were stiffer than normal fibroblasts. Fluorescence images (Fig. 8) clearly show that the cell’s stress fibre network has a strong role in determination of cell stiffness. Interestingly, cell stiffness depends as well on the decellularized matrix composition along with its stiffness. Note that the error bars from Fig. 9 are large, which is due to the fact that force maps were recorded on different cellular regions (nucleus, cell body and periphery), presenting therefore a larger range of Young’s modulus values.

**Table 4:** The median (bold values) Young’s moduli (kPa) values of normal, scar and Dupuytren’s fibroblasts from cell-populated matrix category filtering the matrix modulus values.

Matrix	Amnion			DED			Epiflex			MatriDerm			XenoDerm		
Fibroblast type	25th	Median	75th	25th	Median	75th	25th	Median	75th	25th	Median	75th	25th	Median	75th
normal	0.07	**0.14**	1.21	1.07	**1.49**	2.09	0.14	**0.31**	0.46	0.77	**1.51**	2.21	0.26	**0.36**	0.35
scar	0.60	**0.94**	0.85	3.33	**5.65**	3.29	0.89	**1.31**	4.45	3.76	**6.55**	8.71	0.74	**0.94**	3.47
Dupuytren	1.43	**2.28**	3.22	3.03	**6.03**	3.30	0.68	**1.4**	5.01	3.88	**5.75**	10.71	1.12	**1.58**	3.90

### Fibroblast invasion into the decellularized matrices

**Figure 10:**
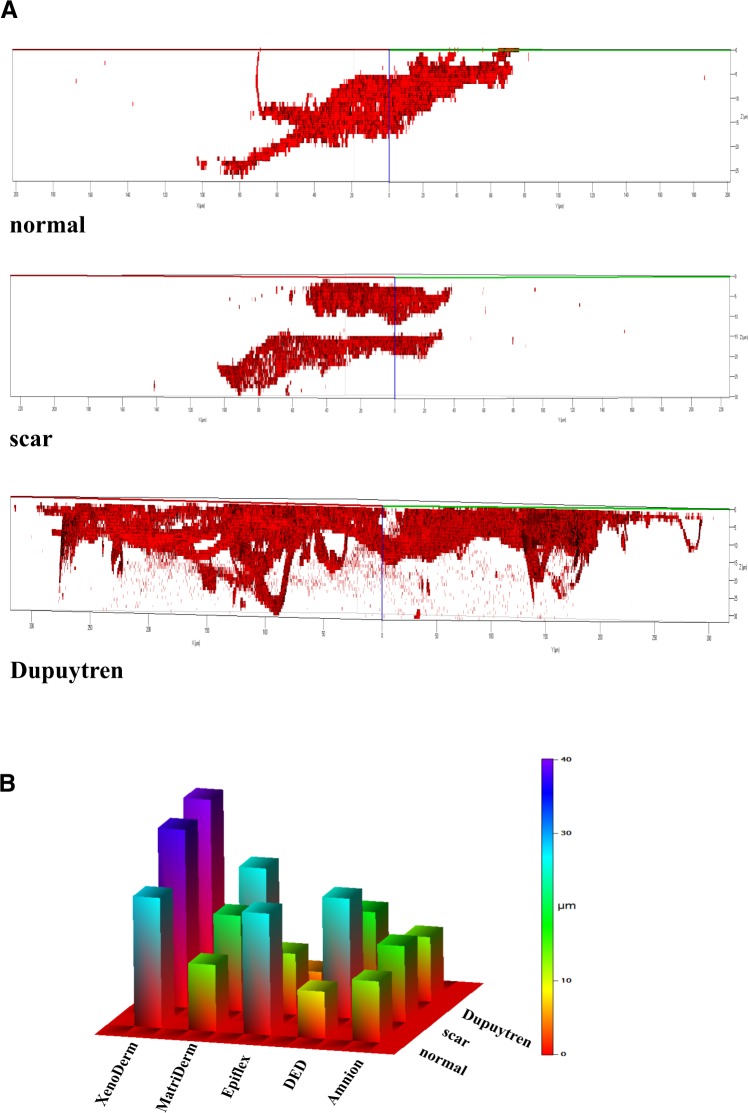
Fibroblast invasion into decellularized matrices, studied with the confocal microscope. (**A**) Representative 3D plots were created from the z stack images and show the invasion of normal, scar and Dupuytren’s fibroblasts into the Amnion decellularized matrix. Pathological (scar and Dupuytren’s) fibroblasts are more invasive than normal fibroblasts into this matrix. The remaining plots of fibroblasts invading into the other matrices are shown in Supplementary Figs 4–7 (**B**) The 3D color category plot extrapolated from Supplementary Fig. 8 represents the value range of normal, scar and Dupuytren’s fibroblast invasion into all five decellularized matrices.

Cellular invasion into a matrix is found both in physiological and pathological conditions. While fibroblasts invade the wound bed and initiate repair processes by synthesizing new matrix components, excessive matrix production is found in various fibrotic diseases like idiopathic pulmonary fibrosis and Dupuytren’s contracture. In malignancies, e.g. scirrhous gastric carcinoma (SGC), stromal fibroblasts mediate the ECM microenvironment mechanical remodeling and the invasion of carcinoma cells^36^. To quantitatively address the invasion of fibroblasts into the decellularized matrices, we acquired confocal z stack images on matrices for normal and pathological (scar and Dupuytren’s) fibroblasts, which were fluorescently labeled for actin with rhodamine phalloidin. Typical 3D plots (Fig. 10A) generated from z stacks taken at different positions on individual matrices show a distinct pattern of invasion for each fibroblast type. This invasive pattern made it possible to distinguish between normal, scar and Dupuytren’s fibroblast in decellularized matrices. Viewing the z-axis (µm) of 3D plots, the degree of invasion by fibroblast can be seen. In Amnion (Fig. 10A), DED (Supplementary Fig. 4), MatriDerm (Supplementary Fig. 6) and XenoDerm (Supplementary Fig. 7), pathological (scar and Dupuytren’s) fibroblasts were more invasive than normal fibroblasts. This contrasted findings with Epiflex (Supplementary Fig. 5) where normal fibroblasts showed higher invasiveness than pathological fibroblasts. The fluorescence intensity versus invasion depth calculated from z stacks were plotted for the three fibroblast cell types (Supplementary Fig. 8A–E). From this plot, a 3D color category plot (Fig. 10B) was generated by calculating the 50th percentile of the invasion depth for every fibroblast cell type on individual matrices. Within the matrices, normal fibroblasts invaded largely in Epiflex and XenoDerm with an invasion depth falling in the range of 20–25 µm and 25–30 µm, respectively. With regard to Amnion, DED and MatriDerm, they invaded with an invasion depth falling in the range of 10–15 µm. Pathological fibroblasts invaded DED and MatriDerm matrices with an invasion depth in the range between 20 µm and 25 µm while invasion of the XenoDerm matrix was very high with a depth in the range of 35 µm and of Amnion and Epiflex matrix with a smaller depth of 5–15 µm. Among all the decellularized matrices, all three fibroblast types showed highest invasiveness in collagen rich XenoDerm matrix. Even in XenoDerm matrix, scar and Dupuytren’s fibroblasts were more invasive than normal fibroblasts. From the AFM PeakForce Tapping images, no micro scale pores were seen on any of the five decellularized matrices. This implies that the presence of fibroblasts inside the matrix or the tendency to infiltrate it depends on the invasive behaviour of the corresponding cell type. These results clearly demonstrate a higher invasive behaviour of pro-fibrotic scar and Dupuytren’s fibroblasts in contrast to normal dermal fibroblasts.

## Discussion

We have demonstrated that different types of fibroblasts isolated from the same patient are capable of remodelling the topography and elasticity of decellularized matrices in different ways. In particular, by obtaining AFM peak force images and mechanical force maps, we showed the interdependency of cell and ECM mechanics in these various decellularized matrices. Z stack images from confocal microscopy provided additional quantitative information on cell invasion into matrices, revealing the variable degree of cell invasion and actin stress fibre formation of fibroblasts in different matrices. Additionally, we showed that cell stiffness strongly varies depending on respective decellularized matrix which has different mechanical and biochemical properties (Fig. 9). In order to follow the cell and ECM mechanics simultaneously, the experimental setup was designed in such a way that the same decellularized matrices were used for obtaining AFM peak force images and mechanical force maps in each category, e. g bare, cell-occupied and cell-removed matrices. This was done to establish the effect of cell culture on matrix topography and mechanics with respect to each fibroblast type (normal, scar and Dupuytren). As these matrices are basically created for wound healing and tissue regeneration applications, we have selected these five matrices in order to investigate ECM maintenance of fibroblasts by topographical and mechanical evaluation. These matrices have different topography, mechanical stiffness and biochemical compositions. Taking into account the presence of collagen in all matrices, there are other varying ECM components present in these matrices. Histochemical and immunofluorescence analysis from earlier studies shows that Amnion contains collagen I, III and IV, laminin, elastin and fibronectin^37^, DED contains collagen I, III, IV, V and VI, other glycosaminoglycans and glycoproteins^28^, Epiflex contains collagen I, III and IV, laminin, fibronectin, vitronectin and hyaluronic acid^35^, MatriDerm contains collagen and elastin^26^ and XenoDerm contains collagen^27^. Mostly, the topographic images of these matrices show the presence of fibres (thick and thin)- which could be both collagen and elastin. These fibres are harder to distinguish biochemically without any histochemical or fluorescence analysis. To favor the aim of this study, these mechanical, topographical and biochemical compositional varying matrices are suitable enough to observe the matrix remodeling by fibroblasts. While the hydration of elastin^38^ and water sequestration by proteoglycans^39^ modify ECM elasticity and swelling, we found that the liquid environment did not change the topography and elasticity of most of our decellularized matrices, at least within a range of 14 days (Fig. 2). A big change in Young’s modulus value was solely found for MatriDerm matrix, even if the elasticity changes due to the liquid environment did not affect matrix topography. Previously, the exploration of cell mechanics and its influence on ECM mechanics was carried out on ECM protein coated polyacrylamide (PA) hydrogels^14,40^ or directly on individual ECM components, mostly collagen^16,18,22^, which limits their mechanical evaluation in *in vivo* environments. Here, we prepared a decellularized human dermal matrix (DED) by the freezing and thawing method^33^. This procedure preserves the ECM mechanical properties^41,42^ and enables measuring direct cell and ECM micromechanics in their natural microenvironment, providing the cells with a complex and variegated amount of ECM signals. Along with other decellularized matrices, these natural scaffolds provide a more physiological support to conduct biological and biophysical experiments on the fundamental reciprocal interplay between cells and ECM.

Cells exert traction forces on their microenvironment which leads to ECM protein realignment through reversible strain stiffening^18^ and as a consequence, ECM fibres enable long range stress transmission between cells^43^. Intracellular actomyosin contractility enables wound closure^44,45^ and increases tissue stiffness^46^. Contractile stress fibres^47^ act as a key element in fibroblast mechanics. Here, we performed mechanical force measurements and correlated our results with confocal microscopic images. Thereby, we could demonstrate that cutaneous scar and palmar fascial Dupuytren’s fibroblasts expressed large stress fibres on all decellularized matrices which contributed to an increase in Young’s modulus values of these cells in contrast to normal fibroblasts. Under the influence of mechanical tension and transforming growth factor-beta, fibroblasts differentiate into myofibroblasts during wound healing or tissue fibrosis^48^. In these environments, they exert larger traction stresses on substrates through expression of alpha smooth muscle actin (α-SMA) stress fibres. Our earlier results showed the existence of a myofibroblast like phenotype in Dupuytren’s fibroblasts that expressed α-SMA positive stress fibres^49^. In case of the dermal scar fibroblasts which previously^49^ showed α-SMA positive but no large stress fibres, we observed the presence of large actin stress fibres in the present experiment. This phenomenon can be explained by the fact that the matrix biophysics governs the activation and differentiation of fibroblasts with transition into a protomyofibroblast/myofibroblast phenotype. These mechanical phenotypic and actin isoform genotypic characteristic differences were observed in normal and pathological (scar and Dupuytren’s) fibroblasts^49^. Another factor that could have contributed to the activation of fibroblasts with conversion into their contractile phenotype is the fact that we plated scar fibroblasts on substrates similar to their native environment in this study. For example, cells are stiffer in their native environment than when plated on glass^50^ or plastic Petri dishes. Despite their phenotypic differences, all three fibroblast types modified the physical structure of the respective decellularized matrix they were seeded onto. Previously, AFM helped to visualise the changes in molecular weight, volume and average height of the ECM protein when coated on a glass coverslip. These measured changes give valuable information on the ECM degradation by cells^17^. Here, AFM PeakForce images of decellularized matrices after removing the cells clearly showed misaligned and aggregated ECM fibres and confirmed the ECM remodeling by cells. In addition, the mechanical stiffness of fibroblasts populated decellularized matrices was analyzed by quantitative force maps. Changes in quantitative force mapping were associated with changes in the matrix mechanical environment when the bare and cell-removed matrices were compared.

Cells sense the mechanical properties of their extracellular matrix by exerting traction forces that are generated by intracellular contractile actin stress fibres. It has been shown that these traction forces linearly contribute to ECM degradation^51^. In accordance, pathological (scar and Dupuytren’s) fibroblasts that express large actin stress fibres contribute to soften their microenvironment to a much lower degree than normal fibroblasts. Pro-fibrotic fibroblasts maintain their contractile behaviour via stiffening their environment which is explicitly seen with MatriDerm. Despite of the fact that the liquid environment influenced the Young’s modulus to the order of 10^3^ Pa in MatriDerm (Fig. 2B), the matrix elasticity increased to the order of 10^4^ Pa after removing both scar or Dupuytren’s fibroblast cell types (Supplementary Fig. 2D). Additionally, we confirmed that 1% trypsin, 0.5% Triton X-100 and 1% SDS that were used for cell removal had no effect on matrix topography and Young’s modulus (Supplementary Fig. 9A,B). This further confirms that changes in topography and mechanics observed in cell-removed matrices are effected by the cells that had been plated onto the matrices. Our results show clearly that contractile myofibroblasts of a dermal scar or palmar fascial Dupuytren’s disease origin continuously remodel the extracellular matrix which results ultimately in matrix stiffening^48^. On the other hand, normal fibroblasts soften their microenvironment largely in order to maintain their quiescent state. Previously, AFM nanoindentation tests on idiopathic pulmonary fibrosis and normal lung tissues showed the stiffer tissue properties in lung fibrosis than in normal lung tissues^52^. Accordingly, our AFM mechanical mapping of pathological (scar and Dupuytren’s) and normal fibroblasts on all decellularized matrices shows exactly the same stiffer and softer tissue properties, respectively. This clearly means that the AFM nanoindentation tests on tissue or cells on decellularized matrices are great for distinguishing between fibrotic or scar and normal tissues. Further exploration on the pathological fibroblasts shows that the cancer associated fibroblasts (CAF) isolated from breast cancer environment promotes matrix stiffening and cancer cell invasion by activating intracellular transcriptional regulators-YAP^53^. Altogether, our results confirm that the fibroblast mechanics reciprocally influence their surrounding microenvironment mechanics.

Our results further indicate that fibroblast stiffness depends on the composition of the decellularized matrices onto which they are seeded. The Young’s modulus values of the three fibroblast types were different on all decellularized matrices, irrespective of matrix stiffness. In this study, the elasticity of most of the decellularized matrices was in the order of 10^5^ Pa, except of the very stiff Amnion. In order to observe the fibroblasts Young’s modulus within the decellularized matrices, we filtered the matrix Young’s modulus values from the cell populated mechanical maps. With varying ECM composition among the decellularized matrices, there was a distinct influence on the fibroblast stiffness. Therefore, we could evaluate the values of cell stiffness in relation to respective decellularized matrix. Previous studies showed that the reorganisation of ECM components altered the tissue elasticity of damaged livers^54^. Obviously, the ECM composition and its stiffness seem to have a direct effect on the regulation and activation of the corresponding cell phenotype^55^. In accordance, despite of the variability of the decellularized matrices’ stiffness and composition, pathological fibroblasts were stiffer than normal fibroblasts in our study. Furthermore, the pathological fibroblasts greatly maintained their proto-myofibroblast/myofibroblast like phenotype and dermal fibroblasts their regular fibroblast like phenotype while invading into these matrices.

Cellular migration on and invasion into tissues are important for physiological wound repair and for diseases like cancer metastasis and fibrosis^56^. With the help of confocal microscopy z stack images, we were able to monitor the degree of fibroblast invasion into the decellularized matrices. Surprisingly, scar and Dupuytren’s fibroblasts colonized the top of the Epiflex matrix and were less invasive than normal fibroblasts. On the other hand, normal, scar and Dupuytren’s fibroblasts were highly invasive into the collagen rich XenoDerm matrix. Cellular lamellipodia extend into the collagen matrix^16^. This conflicting state of fibroblast invasion might be due to the influence of extracellular matrix collagen fibre orientation in cell invasion^57^. Finally, this finding might be important for the clinical application of various matrices. Acellular matrices are currently used for implant coverage in esthetic plastic surgery. Hence, a matrix that prevents fibroblast penetration could also contribute to a reduction of fibrotic capsule formation.

Even if we were unable to assess the influence of ECM protein fibre alignment or cellular integrin expression^58^ on cell invasion by biophysical means, we found that contractile pathological fibroblasts are highly invasive in comparison to normal fibroblasts. The mechanically activated myofibroblasts confirmed their phenotypically expected behaviour by vastly invading the ECM. Under pathological conditions, myofibroblasts show an aggressive and invasive phenotype in tissue fibrosis^59^. Extracellular matrix invasion is enhanced by cell stiffness^60^. In accordance, we found large stress fibres in pathological fibroblasts that were stiffer and invaded the decellularized matrices much more profoundly than normal fibroblasts.

## Conclusion

In conclusion, we have shown that the decellularized matrices are suitable substrates to investigate the interdependent reciprocal interplay between cell and ECM mechanics using AFM force spectroscopy. Pathological fibroblasts were stiffer than their normal counterparts and showed higher invasive behaviour into different matrices with subsequent higher stiffening of the decellularized matrices (specifically MatriDerm) used in our study. Furthermore, we showed that cell stiffness depends not only on cell microenvironment mechanical properties but also on the matrix biochemical composition. These findings have a dual important impact in translational research: (1) they will foster further biophysical studies in the field of tissue engineering, and (2) they provide valuable information to improve commercially available acellular matrices that are currently used in various clinical settings, like tissue regeneration and wound healing.

## Materials and Methods

### Decellularized matrices and DED Preparation

Human acellular amnion and Epiflex acellular dermal matrix were kindly donated by the German Institute for Cell and Tissue Replacement (Deutsches Institut für Zell- und Gewebeersatz, DIZG, Berlin, Germany). Collagen-elastin rich synthetically produced MatriDerm was purchased from Dr. Otto Suwelack Skin &Health Care AG, Billerbeck, Germany. Porcine XenoDerm acellular dermal matrix was purchased from Medical Biomaterial Products (MBP) GmbH, Neustadt-Glewe, Germany.

De-epidermized dermal (DED) matrix was prepared from excised human tissue as described elsewhere^33^. First, tissues were cut and punctured into small circular pieces (diameter ~4 mm and thickness ~2 mm) by using a trephine and the fat layer beneath the tissue was removed with a scalpel. The tissue was then transferred to the 50 mL falcon tubes containing PBS and incubated in a water bath at 56 °C for 30 min. After that, the epidermis was easily stripped off as the upper dark skin layer was identified as epidermal layer and removed with tweezers. The cells in the dermal layer were destroyed by 10 cycles of freezing and thawing. Then the DEDs were stored at −20 °C for future experiments.

### Primary human fibroblast cultures

Tissues for cell harvest were obtained from patient undergoing plastic reconstructive and hand surgery. Scar fibroblasts were derived from scar excision and normal from adjacent skin tissue. Dupuytren’s fibroblasts were isolated from excised nodules and palmar strands of the same patient who presented with Dupuytren’s disease. Patient was informed pre-operatively and had given their informed consent to anonymous tissue donation. The study was approved by the local Ethics Committee (Ärztekammer Bremen, #336/2012). The guidelines of the declaration of Helsinki were followed.

### Fibroblast extraction

For cell culture, the tissue was minced and enzymatically disintegrated using a 0.5% collagenase solution (250 U/ml Serva, Heidelberg, Germany) at 37 °C in 5% CO_2_ for 6 h. After centrifugation, the pellet was resuspended in culture medium (TC 199 with Earle’s salts supplemented with 20% fetal bovine serum, 200 IU/ml penicillin, 200 µg/ml streptomycin) and incubated at 37 °C in 5% CO_2_ air. The culture medium was changed after attachment of the cells. Primary fibroblasts of the three different skin tissues were passaged using trypsin/EDTA solution (0.05%/0.02% w/v in PBS w//o Ca^2+^, Biochrom, Berlin, Germany) a split ratio of 1:2 one time a week to preserve monolayer formation.

### Cell culture on decellularized matrices

Cells were seeded on the decellularized matrices two weeks before AFM or confocal measurements. In brief, decellularized samples were glued to the Petri dish using the super glue (Tesa Sekundenkleber), taking special care to make sure that glue was in contact only to the bottom layer of the matrices without penetrating through the top layers. We used tiny droplets of super glue, to immobilize our matrix samples, which solidifies faster and thus prevents the contact of glue to the top layer of the samples. PBS was added quickly onto the matrix to prevent drying. Prior to cell seeding, the matrices were extensively washed in PBS for 30 min, and incubated for a few hours with DMEM medium, supplemented with 10% fetal bovine serum (FBS) and 2% penicillin–streptomycin. Then cells were seeded in DMEM medium and incubated at 37°C in a humidified atmosphere of 95% air and 5% CO_2_. Cell culture was established for two weeks before proceeding with further measurements. Medium was replenished every three days and supplemented with 10% fetal bovine serum (FBS) and 2% penicillin-streptomycin. Passages between three and eleven were used for all the experiments.

To remove cells from the matrices, the medium was first removed and samples were washed twice with PBS. Then the matrices were treated with 1% trypsin for 3 min and incubated with a solution containing 0.5% Triton X-100 and 1% SDS (sodium dodecyl sulphate) for 5 min at 37 °C. Matrices were washed twice and stored with PBS for AFM imaging and mechanical measurements.

### PeakForce tapping mode Imaging

A Bruker BioScope Resolve AFM (Bruker Nanotechnologies, Santa Barbara, CA) was used to image the matrices, with and without cells. Commercially available cantilevers (PFQNM-LC probes, Bruker, spring constant 0.1 N/m) were used for imaging. Peak Force Quantitative Nano Mechanical imaging for live cells mode was done at oscillation frequency of 1 kHz using a Peak Force Tapping amplitude of 300 nm (for bare and cells removed matrices) and 600 nm (for cells populated matrices) and a peak force of 400 pN. Scanning was performed at a rate of 0.270 Hz. An optical microscope was combined with the AFM to be able to control tip and sample positioning. The AFM head including the sample was enclosed in a homebuilt polymethacrylate (PMMA) box in order to maintain 5% CO_2_ in the atmosphere. Images were recorded with the Nanoscope working software, version 8.15 and image processing was performed with the Nanoscope Analysis software, version 1.8.

### AFM force mapping

A MFP3D AFM (Asylum Research, Santa Barbara, CA, USA) was used to measure mechanical properties of bare, cell-occupied and cell-removed matrices for three types of fibroblasts. An optical microscope (Zeiss Axiovert 135, Zeiss, Oberkochen) was combined with the AFM to be able to control tip and sample positioning. All measurements were performed with the same soft cantilever (MLCT Bio, Bruker, nominal spring constant 0.01 N/m). The Petri dishes with matrix samples were fixed to an aluminum holder with vacuum grease and mounted on the AFM stage with two magnets. The AFM head including the sample was enclosed in a homebuilt polymethacrylate (PMMA) box in order to inject and maintain 5% CO_2_. Force maps were recorded on matrices and living cells on matrices to study their mechanical properties. First, the spring constant of the cantilever was calibrated by using the thermal tune method on a cleaned and stiff surface^61^ and then force curves were recorded. For force curves, we used typically a ramp rate of 1 Hz, corresponding to a maximum loading rate of 1 nN/s and a maximum force of 1nN. Indentation depths were always greater than 500 nm in order to average the stiffness over a large contact area, which gives values that do not depend on local variations of the cytoskeletal or matrix structure. All AFM measurements, imaging and mechanical mapping on matrices after adding cells were performed on living cells.

### AFM data analysis

The data analysis package IGOR (wave metrics, Lake Oswego, OR, USA) was used to evaluate mechanical properties of the cells and decellularized matrices. Details have been described elsewhere^62^. Only approach force curves were analysed within the framework of the Hertz model for pyramidal tips in order to obtain the apparent Young’s modulus of the samples^63–65^. At least a total of 10 force maps were recorded at 10 different positions on matrices for each category. Each force map contained 2500 force curves (50 × 50 lines per frame) over an area of typically 30 µm. The median values of 10 force maps (10 × 2500 = 25000 force curves) was considered as a representative modulus of each category (force maps were recorded over 10–12 different positions for each category).

### Cell young’s modulus analysis from cell populated matrix force maps

To study matrix composition dependent cell stiffness, the Young’s moduli of cells were calculated from the cell populated matrices. Most of the force maps were taken directly on the cell and in exception some maps contains both cell and matrix regions. The slope (Supplementary Fig. 3A) and contact point approach map (Supplementary Fig. 3B) helps to distinguish the matrix and cell regions. The AFM tip contact the sample with definite contact point which can be calculated for each force curve in the approach curve. This contact point reflects the height of the sample from which cell and matrix regions are recognized. The slope color-scale shows the slope values above 0.375 (corresponding force curve shown in Supplementary Fig. 3C3 and in the respective force maps) falls in the matrix and below 0.375 falls in the cell regions. By substituting 0.375 as threshold value, the Young’s modulus values from matrix regions were excluded after careful examination of each force maps. In addition, the force curves obtained in the matrix (Supplementary Fig. 3C1) and cell (Supplementary Fig. 3C2) clearly shows the distinction. The separation of approach and retract curves in the slope area are smaller in forces curves obtained from matrix than in cell region. The force *versus* indentation graph (Supplementary Fig. 3D) shows the larger indentation for cells than the matrix. The separation between approach and retract curves is a measure of the viscosity of the cell, however there is a cross talk with softness of the cell, as we have shown in previous work analyzing the response of step forces of cell^62^. Thus we used here only the slope to distinguish between cell and matrix rather than more sophisticated but derived quantities like elastic modulus or separation of force curves.

### Confocal microscopy and immunofluorescence staining

To study cell invasion into matrices, we used a Zeiss LSM 780 Confocal with 40x oil objective lens. Two weeks after seeding of cells on matrices, cells were fixed with 3.7% formaldehyde for 15 min and permeabilized with 0.1% Triton X100 for 3 min. Samples were washed with PBS after each step and then incubated with a rhodamine phalloidin solution (5:200 dilution in PBS) for F-actin staining for 30 min at 20°C and samples were washed after every step with PBS. Finally, samples were washed and stored in PBS at 4 °C prior to image acquisition. The confocal laser lines 561 nm (excitation) and 570–650 nm (emission) were used for obtaining z stack images of cells stained for actin within the matrices. Z stacks were collected and analysed from 3D stacks using ZEN software version 2.0.

### Statistical analysis

Statistical differences for the median values of Young’s moduli of bare, cell occupied and cell removed matrices of the AFM measurements were determined by Wilcoxon test, calculated in IGOR software. * and ** indicate statistically significant differences for p-values < 0.05 and p < 0.005, respectively.

## Supplementary information

Supplementary Information

## Data Availability

The datasets generated during and/or analysed during the current study are available from the corresponding author on reasonable requests.
