# Supplementary material for: Nano-mechanical mapping of interdependent cell and ECM mechanics by AFM force spectroscopy
Source: Sci Rep. 2019 Aug 23;9:12317. doi: 10.1038/s41598-019-48566-7 (PMC6707266; doi:10.1038/s41598-019-48566-7)
Supplement: Supplementary file 1 — Supplementary Information [file 41598_2019_48566_MOESM1_ESM.docx]

Nano-mechanical mapping of interdependent cell and ECM mechanics by AFM force spectroscopy

Prem Kumar Viji Babu^1^, Carmela Rianna^1^, Ursula Mirastschijski^2^, Manfred Radmacher^1*^

^1^Institute of Biophysics, University of Bremen, Bremen, Germany

^2^Wound Repair Unit, Centre for Biomolecular Interactions Bremen, University of Bremen, Bremen, Germany

*To whom correspondence should be addressed. E-mail: mr@biophysik.uni-bremen.de

**Supplementary Information**

**Supplementary figure 1** (A) The high resolution map showing the ECM fibers (blue arrows) running along the Amnion decellularized matrix. (B) The force map recorded on the Amnion matrix shows 50 x 50 force curves and the white areas indicates the stiff areas and the black spots indicates the soft areas. Force curve from stiff (1) and soft (2) areas within the Amnion matrix were analyzed and the Hertz Fit window for Young’s modulus calculation are shown, respectively.

**Supplementary figure 2** The median of Young’s modulus values of the bare (native, black), cell-populated (blue) or cell-removed (red) decellularized matrices for normal, scar and Dupuytren’s fibroblasts were plotted for each matrix, i.e. Amnion (A), DED (B), Epiflex (C), MatriDerm (D) and XenoDerm (E). The respective median of Young’s moduli values are presented in Table 1, 2 and 3. Statistical results are reported in Materials and Methods section.

**Supplementary figure 3** Extrapolation of cell Young’s modulus from cell populated matrix. The slope (A) and contactpoint approach (B) map shows the distinctive cell and matrix region. The force curves (C) of the matrix [1] and cell [2] region showing separation of approach and retract curves which is smaller in matrix and comparatively larger in cell. The force curve from the matrix region [3] of the force map corresponds to slope value 0.3748. (D) Force *vs* indentation graph (full line) with Hertz fit (discontinuous line) shows the two slope values of matrix (0.4186) and cells (0.1546). The threshold slope value (0.375) was used as a critical value to neglect the matrix contribution to cell stiffness, after careful analysis of cell-populated mechanical maps.

**Supplementary figure 4** Cell invasion 3D plot on DED matrix. The 3D plot was created from the z stack images recorded by confocal microscopy. Scar and Dupuytren’s fibroblasts were more invasive than normal fibroblasts into the DED matrix.

**Supplementary figure 5** Cell invasion 3D plot on Epiflex matrix. The 3D plot created from the z stack images shows that normal fibroblasts penetrate more than pathological (scar and Dupuytren’s) fibroblasts into the Epiflex matrix.

**Supplementary figure 6** Cell invasion 3D plot on MatriDerm matrix. The 3D plot was created from the z stack images and shows the invasion of normal, scar and Dupuytren’s fibroblasts into the MatriDerm matrix.

**Supplementary figure 7** Cell invasion 3D plot on XenoDerm matrix. The 3D plot was created from the z stack images and shows the higher invasive tendency of normal, scar and Dupuytren’s fibroblasts into the XenoDerm matrix. With regard to cell types, scar and Dupuytren’s fibroblasts were more invasive than normal fibroblasts.

**Supplementary figure 8** Fluorescence intensity was plotted versus invasion depth from z stack fluorescence images and shows the degree of normal, scar and Dupuytren’s fibroblast invasion into Amnion (A), DED (B), Epiflex (C), MatriDerm (D) or XenoDerm (E).

**Supplementary figure 9** Effect of trypsin, Triton X-100 and SDS on XenoDerm decellularized matrix. (A) Height and PeakForce Error images from AFM PeakForce Tapping and (B) median of Young’s modulus values obtained from mechanical force maps before and after treating the matrix with 1% trypsin, 0.5% triton X-100 and 1% SDS. AFM images and Young’s modulus plot show that there is no significant effect on XenoDerm’s topography and elasticity, respectively.
